# Clinical Outcome and Genetic Differences within a Monophyletic Dengue Virus Type 2 Population

**DOI:** 10.1371/journal.pone.0121696

**Published:** 2015-03-26

**Authors:** Hapuarachchige Chanditha Hapuarachchi, Rachel Choon Rong Chua, Yuan Shi, Tun Lin Thein, Linda Kay Lee, Kim Sung Lee, David Chien Lye, Lee Ching Ng, Yee Sin Leo

**Affiliations:** 1 Environmental Health Institute, National Environment Agency, 11 Biopolis Way, #06-05-08, Singapore 138667; 2 Institute of Infectious Diseases and Epidemiology, Tan Tock Seng Hospital, 11 Jalan Tan Tock Seng, Singapore 308433; 3 School of Life Sciences and Chemical Technology, Ngee Ann Polytechnic, 535 Clementi Road, Singapore 599489; University of Minnesota, UNITED STATES

## Abstract

The exact mechanisms of interplay between host and viral factors leading to severe dengue are yet to be fully understood. Even though previous studies have implicated specific genetic differences of Dengue virus (DENV) in clinical severity and virus attenuation, similar studies with large-scale, whole genome screening of monophyletic virus populations are limited. Therefore, in the present study, we compared 89 whole genomes of DENV-2 cosmopolitan clade III isolates obtained from patients diagnosed with dengue fever (DF, n = 58), dengue hemorrhagic fever (DHF, n = 30) and dengue shock syndrome (DSS, n = 1) in Singapore between July 2010 and January 2013, in order to determine the correlation of observed viral genetic differences with clinical outcomes. Our findings showed no significant difference between the number of primary and secondary infections that progressed to DHF and DSS (p>0.05) in our study cohort. Despite being highly homogenous, study isolates possessed 39 amino acid substitutions of which 10 substitutions were fixed in three main groups of virus isolates. None of those substitutions were specifically associated with DHF and DSS. Notably, two evolutionarily unique virus groups possessing C-P43T+NS1-S103T+NS2A-V83I+NS3-R337K+ NS3-I600T+ NS5-P136S and NS2A-T119N mutations were exclusively found in patients with DF, the benign form of DENV infections. Those mutants were significantly associated with mild disease outcome. These observations indicated that disease progression into DHF and DSS within our patient population was more likely to be due to host than virus factors. We hypothesize that selection for potentially less virulent groups of DENV-2 in our study cohort may be an evolutionary adaptation of viral strains to extend their survival in the human-mosquito transmission cycle.

## Introduction

Dengue fever is the most widespread arbovirus disease at present with an annual estimate of 50 million infections worldwide [1]. The disease is caused by Dengue virus (DENV) complex that consists of four genetically and immunogenically distinct serotypes (DENV-1 to DENV-4). Most DENV infections are benign and manifest either sub-clinically or as a flu-like illness known as dengue fever (DF). However, approximately 500,000 infections each year result in severe disease associated with hemorrhagic manifestations (dengue hemorrhagic fever, DHF) and shock (dengue shock syndrome, DSS), which may lead to fatal complications [1].

DENV is a single stranded positive sense RNA virus with a 11.8 kb genome flanked by two untranslated regions (5’ and 3’ UTRs) and a single coding region for three structural: capsid (C), the precursor of membrane (prM) and envelope (E); and seven non-structural (NS) proteins: NS1, NS2A, NS2B, NS3, NS4A, NS4B and NS5 [2]. Being a RNA virus, DENV undergoes a rapid evolutionary process [3, 4], generating substantial genetic diversity (genotypes) within each serotype [4]. The evolutionary process of DENV selects for strains with enhanced adaptation, resulting in mutant variants that differ in their ability to spread and cause disease [3, 5]. All four DENV serotypes are capable of causing severe dengue clinically [6]. Even though the clinical outcome of DENV infections is dependent on host and viral factors, the mechanism of their interplay leading to severe disease is not fully understood. Previous studies implicated host genetics [7, 8], immune response [9], infection status and viremia levels [10, 11] as predisposing factors for severe dengue. However, role of virus genetics in determining the virulence of natural virus populations is sparsely characterized.

One of the classical examples of genotype-based severity of dengue is the transition from mild to severe dengue outbreaks subsequent to emergence of a DENV-2 genotype of Asian origin in Latin and central America [12]. According to Holmes and Burch (2000), disease severity could be even strain-dependent [3]. While a number of studies indicate specific viral genetic differences that correlate with clinical severity [13–17] and virus attenuation [18], their interpretations to support the strain-dependent concept of virulence are limited by small sample sizes, utilization of relatively short genome sequences, inadequate clinical data and comparison of heterotypic (inter-genotype or inter-serotype) virus populations. As such, there is a need for studies that utilize large-scale, whole genome screening of monophyletic virus populations obtained from well-characterized clinical cohorts. The analysis of monophyletic virus populations is salient as individual isolates of such populations share extremely high genetic similarity, so that interpretations on the association between specific genetic differences and clinical outcomes become more straightforward. In the present study, we, therefore, compared 89 whole genomes of a monophyletic population of DENV-2 obtained from patients with DF, DHF and DSS manifestations in Singapore, in order to determine the correlation between virus genetic differences and clinical outcome.

## Materials and Methods

### Ethics statement

This study was part of the Early Dengue Infection and Outcome (EDEN) and Prospective Adult Dengue (PADS) studies under the STOP Dengue Translational Clinical Research Flagship Program. The National Healthcare Group Domain Specific Review Board approved the study (DSRB E/05/013, DSRB E/09/432).

### Sample collection

The study included 89 patients infected with DENV-2 between July 2010 and January 2013 in Singapore during which DENV-2 was the dominant serotype [19]. In overall, DENV-2 contributed to more than 70% of serotyped cases in Singapore from 2007 to 2011 [19]. All patients were prospectively recruited upon obtaining written informed consent. Serum/plasma samples were obtained from each patient during the acute phase, and their clinical and demographic information was recorded using standardized data collection forms as described elsewhere [20–22].

### Definition of clinical categories and infection status

DENV infections were classified as DF, DHF and DSS based on the World Health Organization (WHO) 1997 guidelines [23]. Patients in DHF and DSS categories had all of fever, thrombocytopenia, plasma leakage and evidence of hemorrhagic manifestations. Moreover, patients suffering from DSS had tachycardia with narrow pulse pressure, or hypotension for age in addition to signs of hemorrhage. All remaining DENV infections that did not fulfil DHF and DSS criteria were classified as having DF (mild disease). Primary and secondary infection statuses were determined based on the detection of serum IgM and IgG in patient sera, by using Panbio Dengue Capture IgM ELISA and Dengue Capture IgG and Indirect IgG ELISA kits (Alere Inc., Queensland, Australia) respectively. The definitions of primary and secondary infections are described elsewhere [20].

### Detection, isolation and sequencing of DENV-2

#### Detection of DENV-2

Viral RNA was extracted from patient sera/plasma by using the QIAamp Viral RNA Mini Kit (Qiagen, Hilden, Germany) according to manufacturer’s instructions. Detection and serotyping of DENV was achieved by amplifying viral RNA using a quantitative real-time polymerase chain reaction (PCR) protocol described previously [24]. Crossing point values from the real-time PCR assay results were used as a surrogate indicator of virus titres as previously described [24]

#### Isolation of DENV-2

DENV was isolated from sera/plasma using the *Ae*. *albopictus* clone C6/36 mosquito cell line (ATCC CRL-1660). Briefly, the monolayer of C6/36 was inoculated with serum in Leibowitch L-15 medium (Life Technologies, Carlsbad, USA) supplemented with 3% FCS at 33°C to allow for virus adsorption and replication. The infected fluid was harvested after 4–8 days. The presence of DENV in cell supernatants was confirmed by an immunofluorescent assay (IFA) using DENV group-specific and serotype-specific monoclonal antibodies derived from hybridoma cultures (ATCC: HB112, HB114, HB47, HB46, HB49 and HB48). The fluorescein isothiocyanate-conjugated goat anti-mouse antibody was used as the detector. A maximum of two passages was performed for each sample based on the original virus load.

#### Whole genome sequencing of DENV-2

All virus strains isolated from patient sera were subjected to whole genome sequencing. RNA was reverse transcribed using the Superscript III First-strand Synthesis System (Life Technologies, Carlsbad, USA). Each DENV-2 whole genome was amplified in 6 fragments, together with 5’ and 3’ untranslated regions (UTRs) using the Phusion Flash High-Fidelity PCR Master Mix (Thermo Fisher Scientific Inc., Pittsburgh, USA) as described elsewhere [25]. Amplified products were visualized in 2% agarose gels stained with GelRed (Biotium Inc., CA, USA) and were subsequently purified by using Expin PCR SV (GeneAll Biotechnology, Seoul, Korea). Sequencing was performed at a commercial facility according to the BigDye Terminator Cycle Sequencing kit (Life Technologies, Carlsbad, USA) protocol. Sequences were deposited in Genbank database under the accession numbers from KM279513 to KM279610.

### Analysis of sequences

Contiguous sequences were assembled using the Lasergene package version 8.0 (DNASTAR Inc., Madison, WI, USA). The resulting sequences were aligned in the BioEdit Sequence Alignment Editor version 7.0.9.0 [26].

#### Phylogenetic analysis

The phylogeny of study sequences was inferred by the maximum-likelihood (ML) method based on the General Time Reversible (GTR) substitution model and gamma distributed rates with invariant sites in MEGA 6.06 [27]. The robustness of the original tree was tested with 1000 bootstrap replications. Comparison of amino acid sequences of study isolates with those reported in NCBI database was performed by using the BioEdit Sequence Alignment Editor version 7.0.9.0 [26] software to identify unique mutations in study isolates. The median joining network was constructed using the whole genome sequences in Network version 4.6.1.2 [28].

#### Tests for selection pressure

Genome-wide dN/dS ratios were computed using Hyphy package in the Datamonkey web server [29]. The single likelihood ancestor counting (SLAC), fixed effects likelihood (FEL), internal fixed effects likelihood (IFEL) and the mixed effect model of evolution (MEME) methods [30] were used to detect the molecular signatures of selection within an alignment of 272 complete coding sequences consisting of all study isolates (n = 89) and those obtained from GenBank database. The analysis included sequences representing cosmopolitan, Asian, American Asian and American genotypes of DENV-2. Significance levels were set at p<0.05.

#### Secondary structure analysis of UTRs

The secondary structures of the variable region of 3’ UTR and complete 5’ UTR of wild type (NS2A-V83) and mutant (NS2A-V83I) variants of DENV-2 cosmopolitan clade III were predicted using the mfold web server at http://mfold.rna.albany.edu/?q=mfold under standard conditions (37°C) [31].

### Statistical analysis

The statistical significance of the association of primary and secondary infections within the DHF and DSS categories was calculated using the exact binomial test. Student’s t-test was used to calculate the significance of age, number of days of fever at presentation and crossing point values (virus titres) between DF and DF/DSS categories. Student’s t-test was also used to determine any statistically significant differences of crossing point values (viral titres) between mutants and non-mutants.

In order to determine relationships among the three variables; substitutions, clinical outcome (DF and DHF/DSS) and primary/secondary infection status, we first used Chi-squared and Fisher’s exact tests under the null hypothesis that each variable is independent from one another. The data were organized into 2-way tables to estimate the association between any two variables as compared to the third variable. Next, in order to understand the association between individual mutants and clinical outcome in both primary and secondary infections, we calculated relative odds of the occurrence of severe clinical outcome (DHF/DSS) upon exposure to a specific mutant compared to non-mutants using the Cochran-Mantel-Haenszel test. In parallel, the confounding effect of primary/secondary infection in clinical outcome was also addressed through the Breslow and Day test. These two tests are widely used to test the strength of association after controlling for the observed confounders. The data was stratified into multiple 2×2×2 tables by the defined confounders. Odds ratio was calculated to estimate the increased/decrease odds of the pre-defined outcome given the condition of any mutant compared to non-mutants. All the statistical analyses were carried out in R software package version 2.15 [32]. Probability values less than 0.05 were considered statistically significant.

## Results

Of 89 patients recruited, 58 patients developed DF and the remaining developed either DHF (n = 30) or DSS (n = 1). Of them, 45 patients had secondary dengue infections and 27 patients had primary infections [20]. The primary/secondary status of 17 patients could not be classified. Mean age of patients (p = 0.027) and mean number of fever days at presentation (p = 0.02) was significantly higher in DHF/DSS than DF patients (Table 1). Nevertheless, there was no significant difference (p = 0.559) between the primary and secondary infections that progressed to DHF and DSS (Table 1). Crossing point (cp) values, used as a surrogate indicator of virus titres, did not differ significantly (p = 0.41) between DF and DHF/DSS cases (Table 1).

**Table 1:** Summary of the patient and infection characteristics.

Category	DF (n = 58)	DHF/DSS (n = 31)	Statistical significance
Mean age (years)	34.3 (range: 18–66)	39.5 (range: 20–67)	p = 0.027
Mean number of days of fever at presentation	4.2 (range: 1–7)	4.9 (range: 2–7)	p = 0.020
Infection status: Primary	18 (31.0%)	9 (29.0%)	p = 1*
Infection status: Secondary	27 (46.6%)	18 (58.1%)	p = 1*
Median crossing point (cp) value^§^	26.67 (std: 4.2, range: 16.19–34.37)	25.95 (std: 4.35, range: 16.43–31.36)	p = 0.41

The percentages shown within brackets are the proportion of primary and secondary infections in respective clinical categories (DF and DHF/DSS).

*Exact binomial test was used to calculate any differences in association of either primary or secondary infection status with clinical outcome. Remaining p-values shown in the table were calculated using the Student’s t-test.

^§^Crossing point values were generated from the real-time quantitative PCR assay for DENV serotyping and were used as a surrogate marker of virus titres in patient sera [24]. DF = dengue fever; DHF = dengue hemorrhagic fever; DSS = dengue shock syndrome; std = standard deviation

**Fig 1:**
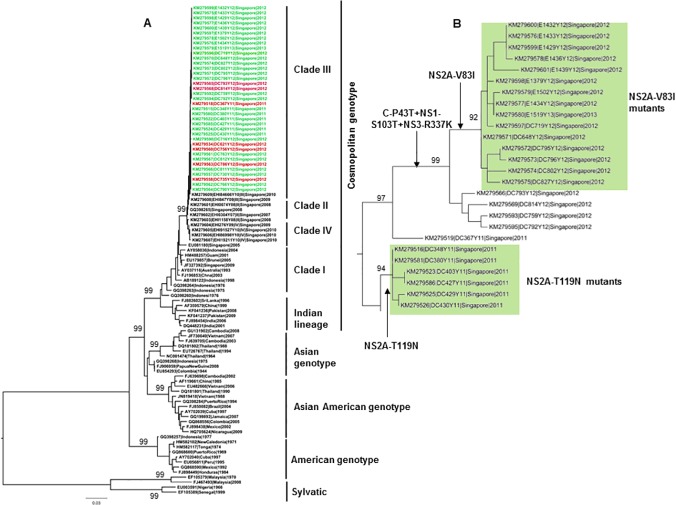
Phylogenetic analysis of DENV-2 study sequences. The maximum-likelihood tree was constructed based on the complete coding sequences generated during this study and those retrieved from GenBank. Sequences of only 37 isolates, mainly comprising of three groups of isolates (NS2A-V83I, n = 15; NS2A-T119N, n = 6; E-V164I+NS2A-L153S, n = 9), are shown in the tree. Viral sequences obtained from patients with DF (mild) and DHF/DSS (severe) manifestations are highlighted in green and red respectively. An enlarged view has been inserted to illustrate the two mutant groups (NS2A-V83I and NS2A-T119N) that were exclusively observed in patients with mild disease (DF) (highlighted in green boxes). Figures on branches are bootstrap values. Only bootstrap values more than 90% are shown on the major nodes.

Envelope (*E*) gene-based phylogeny revealed that 89 virus strains isolated from patient sera belonged to a monophyletic group (clade III) of cosmopolitan genotype (Fig. 1). At complete coding genome level, our study isolates (n = 89) were highly homogenous, sharing 99.5–100% nucleotide and 99.7–100% amino acid similarity. Nevertheless, there were 39 amino acid substitutions distributed among them (S1 Table). Twenty nine substitutions were found either in individual (n = 20) or in groups of 2–3 isolates (n = 9) (Table 2). The remaining 10 substitutions (C-P43T, E-V164I, NS1-S103T, NS2A-V83I, NS2A-L153S, NS2A-T119N, NS3-I600T, NS3-R337K, NS5-P136S and NS5-N645D) were found in three main groups ranging from six to 20 isolates (S1 Table).

**Table 2:** Distribution of amino acid substitutions within DENV-2 cosmopolitan clade III cohort.

Protein	Protein position	Ref.*	Wild type clade III	Mutant clade III	No. of isolates	DF/DHF/DSS	Selection Pressure analysis			
							SLAC	FEL	IFEL	MEME
C	43	P	P	T	19	DF(17),DHF (1),DSS (1)	Neg(0.05)	Neg(0.001)	Neg(0.03)	0.67
C	82	R	R	K	1	DF(1)	Neutral	Neutral	Neutral	1
E	36	K	K	R	1	DHF(1)	Neg(0.03)	Neg(0.01)	Neg(0.01)	0.06
E	164	I	V	I	9	DF(5),DHF(4)	Neutral	Neg(0.02)	Neutral	0.67
E	197	V	V	A	1	DHF(1)	Neutral	Neg(0.02)	Neg(0.02)	Episodic(0.03)
E	250	V	V	I	1	DHF(1)	Neg(0.02)	Neg(0.02)	Neg(0.02)	0.06
E	322	I	I	V	1	DF(1)	Neutral	Neutral	Neutral	0.43
E	347	V	V	A	3	DF(1),DHF(2)	Neutral	Neutral	Neutral	0.41
E	364	P	P	T	1	DHF(1)	Neutral	Neutral	Neutral	Episodic(0.01)
E	428	V	V	M	2	DF(2)	Neutral	Neutral	Neutral	0.12
NS1	40	S	S	P	2	DF(1),DHF(1)	Neutral	Neutral	Neutral	0.13
NS1	103	S	S	T	19	DF(17),DHF(1),DSS(1)	Neg(0.04)	Neg(0.02)	Neg(0.03)	0.67
NS1	286	V	V	A	3	DF(1),DHF(2)	Neutral	Neutral	Neutral	0.06
NS2A	42	T	T	I	1	DF(1)	Neutral	Neutral	Neutral	Episodic(0.03)
NS2A	53	L	L	V	1	DF(1)	Neg(0.04)	Neg(0.002)	Neg(0.006)	Episodic(0.01)
NS2A	83	V	V	I	15	DF(15)	Neg(<0.001)	Neg(<0.001)	Neg(0.005)	0.67
NS2A	113	Q	Q	H	1	DF(1)	Neutral	Neutral	Neutral	0.08
NS2A	118	E	E	K	1	DF(1)	Neutral	Neutral	Neg(0.03)	0.67
NS2A	119	T	S	N	6	DF (6)	Neutral	Neutral	Neutral	Episodic (<0.001)
NS2A	153	L	L	S	10	DF(5),DHF(5)	Neutral	Neg(0.04)	Neutral	0.66
NS3	170	K	K	E	2	DF(2)	Neutral	Neutral	Neutral	0.05
NS3	171	S	S	N	1	DF(1)	Neutral	Neutral	Neutral	0.33
NS3	337	R	R	K	20	DF(17),DHF(2), DSS(1)	Neutral	Neutral	Neutral	0.53
NS3	558	A	A	V	3	DF(2),DHF(1)	Neutral	Neutral	Neutral	Episodic(0.02)
NS3	600	I	I	T	4	DF(4)	Neutral	Neutral	Neutral	0.67
NS5	136	P	P	S	4	DF(4)	Neutral	Neutral	Neutral	Episodic(0.03)
NS5	169	E	E	D	1	DF(1)	Neutral	Neutral	Neutral	0.10
NS5	196	A	A	T	2	DF(1),DHF(1)	Neutral	Neutral	Neutral	0.67
NS5	548	K	K	E	1	DF(1)	Neutral	Neutral	Neutral	0.08
NS5	583	P	P	Q	1	DHF(1)	Neutral	Neg(0.01)	Neg(0.01)	0.67
NS5	645	N	N	D	3	DF(2),DSS(1)	Neutral	Neutral	Neutral	0.29
NS5	648	A	I	T	1	DF(1)	Neutral	Neutral	Neutral	0.62
NS5	725	V	V	A	1	DHF(1)	Neg(0.007)	Neg(<0.001)	Neg(0.002)	0.14
NS5	763	S	S	N	1	DF(1)	Neutral	Neutral	Neutral	0.05
NS5	778	A	A	S	3	DF(3)	Neg(<0.001)	Neg(<0.001)	Neg(0.008)	0.67
NS5	808	D	D	N	1	DF(1)	Neutral	Neutral	Neutral	Episodic(0.02)
NS5	829	P	P	L	1	DF(1)	Neutral	Neutral	Neutral	0.06
NS5	832	S	S	T	3	DF(1),DHF(2)	Neutral	Neutral	Neutral	0.67
NS5	891	R	R	I	1	DHF(1)	Neutral	Neutral	Neutral	0.67

Number of DF, DHF and DSS cases with respective substitutions is shown within brackets in the respective column. The numbers shown under the selection pressure analysis section are probability values. C = Capsid, DF = Dengue fever, DHF = Dengue hemorrhagic fever, DSS = Dengue shock syndrome, E = Envelope, FEL = fixed effects likelihood, IFEL = internal fixed effects likelihood, MEME = mixed effect model of evolution, Neg = Negative selection, NS = Non-structural, SLAC = single likelihood ancestor counting

*Reference sequence = NCBI accession No. NC001474.

The largest group (n = 20) possessed NS3-R337K substitution. There were six additional substitutions fixed in the NS3-R337K group; C-P43T (n = 19), NS1-S103T (n = 18), NS2A-V83I (n = 15), NS3-I600T (n = 4), NS5-P136S (n = 4) and NS5-N645D (n = 3). It was noteworthy that all substitutions (C-P43T+NS1-S103T+NS2A-V83I+NS3-R337K+ NS3-I600T+ NS5-P136S), except NS5-N645D, were detected in NS2A-V83I mutants. While C-P43T+NS1-S103T+NS2A-V83I+NS3-R337K signature was fixed, NS3-I600T (n = 4) and NS5-P136S (n = 4) substitutions were detected only in two separate subsets of NS2A-V83I mutants. In addition, NS2A-V83I mutants (n = 15) possessed two fixed nucleotide substitutions in UTRs (5’UTR- T34C and 3’UTR-A19T). The second largest group possessed NS2A-L153S substitution (n = 10) of which nine isolates shared an additional substitution in the E protein (E-V164I). The third group (n = 6) was characterized by a substitution fixed in the NS2A protein (NS2A-T119N).

None of the mutants described above (either individually or in combinations) showed virus titres that were statistically different from those of non-mutants (data not shown). However, there was a statistically significant association between the clinical outcome and substitutions observed in our study (X^2^ = 39.016, df = 15, chi-squared and fishers’ exact p-values = <0.001), suggesting that observed substitutions had an effect on the clinical outcome of infections. There was no such association between the clinical outcome and primary/secondary infection status (chi-squared p-value = 0.30, fishers’ exact p-value = 0.26) and substitutions and primary/secondary infection status (chi-squared p-value = 0.96, fishers’ exact p-value = 0.98).

**Fig 2:**
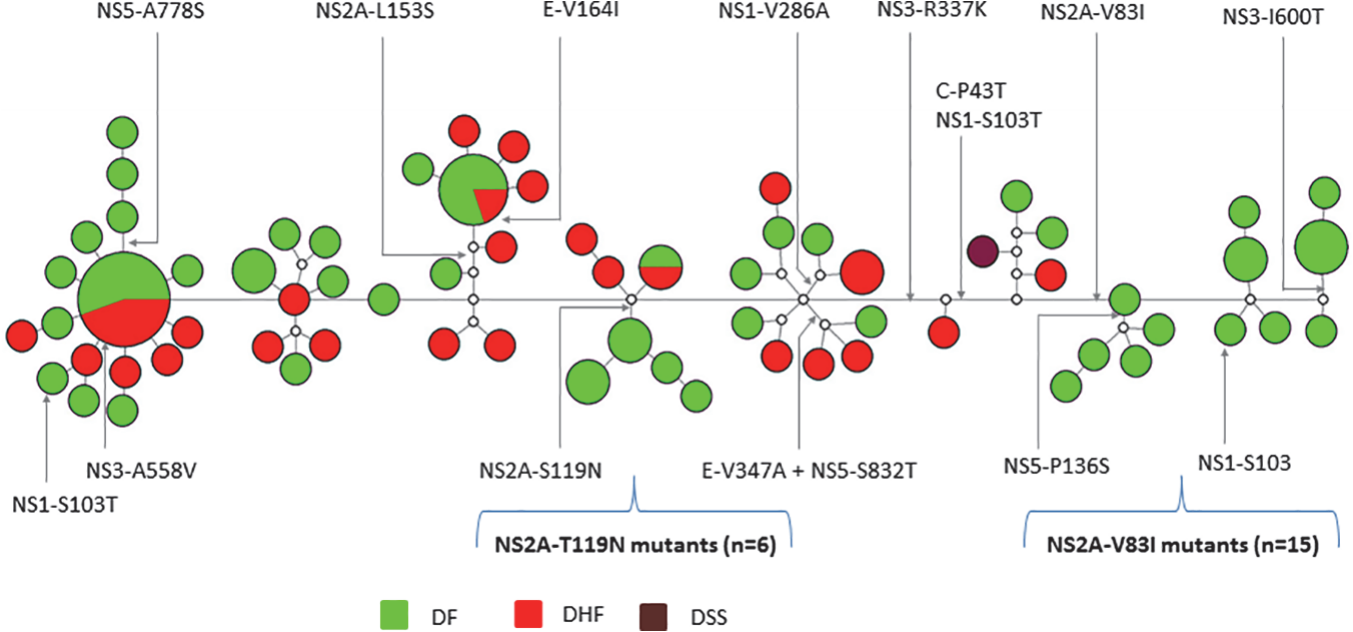
Median joining network analysis and amino acid substitution map of study sequences. The median joining network was drawn in Network version 4.6.1.2 [28] using complete coding sequences (10173 nucleotides) of DENV-2 cosmopolitan clade III sequences (n = 89). Circles represent either individual isolates or clusters. The diameter of each circle is proportional to the number of isolates within each circle. The length of lines linking circles is not proportional to the mutational distance between them. The empty nodes represent hypothetical ancestral strains or strains present in the population but not sampled. Different colors indicate clinical categories as shown in the figure legend. As the analysis only included clade III sequences, mutations were stated as compared to wild-type clade III sequences (Table 2). Only amino acid substitutions associated with groups of isolates (n>6) are mapped. DF = dengue fever; DHF = dengue hemorrhagic fever; DSS = dengue shock syndrome

The analysis of clinical outcome due to infections with the above groups of isolates showed that there were 17 DF and three DHF/DSS cases within the NS3-R337K group. Of them, DF cases were shared equally between primary (n = 9) and secondary (n = 8) infections, but all DHF/DSS cases were secondary infections (S1 Table). Interestingly, neither the 10 substitutions distributed among three main groups of isolates nor those detected in small groups (E-V347A, NS1-V286A, NS3-A558V and NS5-S832T) showed any specific association with severe clinical outcome (DHF/DSS) (Fig. 2). In order to determine which mutations showed the strongest effect on clinical outcome, we calculated relative odds of the occurrence of severe clinical outcome (DHF/DSS) upon exposure to a specific mutant or a combination (haplotype) compared to non-mutants by using Cochran-Mantel-Haenszel and Breslow and Day tests. The analysis showed that isolates and haplotypes with C-P43T (p-value = 0.01), NS1-S103T (p-value = 0.01), NS2A-V83I (p-value = <0.001), NS3-R337K (p-value = 0.01) and NS2A-S119N (p-value = 0.02) substitutions showed significantly low odds of developing severe dengue regardless of either primary or secondary infection status (Table 3). Moreover, the association of NS3-I600T (p-value = 0.06) and NS5-P136S (p-value = 0.07) substitutions with less severe disease was marginally significant (Table 3). Not surprisingly, NS2A-V83I (n = 15) and NS2A-T119N (n = 6) mutants that shared above substitutions were detected only among patients who developed DF (Fig. 2). On the other hand, the association between genetic differences and clinical outcome of isolates (n = 10) possessing E-V164I and NS2A-L153S as well as small groups with E-V347A, NS3-A558V and NS5-S832T substitutions was statistically insignificant (Table 3). Therefore, our findings highlighted that NS2A-V83I and T119N mutants were significantly associated with mild disease (DF) outcome.

**Table 3:** Association between substitutions and the severe clinical outcome (DHF/DSS).

**Substitution**	**Breslow and Day test (with Tarone correction)**		**Mantel-Haenszel Chi-squared test**		
	**X** ^2^ **(df = 1)**	**p-value**	**X** ^2^ **(df = 1)**	**p-value**	**Odds ratio**
C-P43T	2.45	0.12	7.51	0.01	0.13 (0.03–0.65)
E-V164I	0.32	0.57	0.12	0.73	0.76 (0.16–3.57)
E-V347A	1.05	0.3	0.24	0.63	1.82 (0.16–21.07)
NS1-S103T	2.83	0.09	7.51	0.01	0.13 (0.03–0.66)
NS2A-L153S	0.02	0.88	0.00E+00	1	1 (0.23–4.33)
NS2A-S119N	NA	NA	5.11	0.02	NA
NS2A-V83I	NA	NA	10.62	<0.001	NA
NS3-A558V	3.48	0.06	0.3	0.58	0.53 (0.05–5.54)
NS3-I600T	NA	NA	3.67	0.06	NA
NS3-R337K	3.33	0.07	6.02	0.01	0.19 (0.05–0.78)
NS5-A778S	NA	NA	2.4	0.12	NA
NS5-N645D	3.48	0.06	0.3	0.58	0.53 (0.05–5.54)
NS5-P136S	NA	NA	3.39	0.07	NA
NS5-S832T	1.05	0.3	0.24	0.63	1.82 (0.16–21.07)

Relative odds of the occurrence of severe clinical outcome (DHF/DSS), given exposure to a specific mutant compared to non-mutants were calculated using the Cochran-Mantel-Haenszel test. In parallel, the confounding effect of primary/secondary infection in clinical outcome was addressed through the Breslow and Day test. Odds ratio was calculated to estimate the increased/decrease odds of the pre-defined outcome given the condition of any mutant compared to non-mutants. Data is not available (NA) for substitutions that were present only in isolates from DF cases. Odds ratio could not be calculated for NS1-V286A substitution as all DF and DHF cases due to this mutant were secondary infections.

On the other hand, we observed several additional amino acid substitutions in viruses isolated from DHF (E-K36R, E-V197A, E-V250I, E-P364T, NS5-P583Q, NS5-V725A and NS5-R891I) and DF (C-R82K, E-I322V, E-V428M, NS2A-T42I, NS2A-L53V, NS2A-Q113H, NS2A-E118K, NS3-K170E, NS3-S171N, NS5-E169D, NS5-K548E, NS5-A648T, NS5-S763N, NS5-D808N and NS5-P829L) cases. Even though there was an apparent dominance of above substitutions in E and NS2A proteins in DHF and DF cases respectively, it was not able to determine any potential role of those substitutions in clinical outcome because most of them were detected in individual infections.

The comparative whole genome analysis between NS2A-V83I mutants and those reported in GenBank database revealed that C-P43T, NS1-S103T, NS3-R337K and NS5-P136S substitutions were novel. Moreover, NS2A-V83I substitution was found only in two records of American Asian genotype (FJ850060 and FJ873808) from Nicaragua and was unique within the cosmopolitan genotype. Similarly, NS3-I600T substitution has been reported in only one isolate from Puerto Rico (EU482720). Nucleotide positions of 3’UTR-A19 and 5’UTR-T34 were extremely conserved residues in DENV-2 cosmopolitan clade III isolates, except for NS2A-V83I mutants. Similarly, the residue NS2A-119 is highly conserved with serine in all DENV-2 cosmopolitan clade III isolates reported before this study. The high bootstrap support gained by both NS2A-V83I (92%) and NS2A-T119N (94%) mutants in the phylogenetic analysis attested to their genetic distinction (Fig. 1). Moreover, our selection pressure analysis revealed that residues C-43, NS1-103 and NS2A-83 were under strong purifying selection (Table 2), meaning that the evolutionary process of DENV-2 was generally conservative at respective amino acid positions. All those observations indicated that individual isolates within both NS2A-V83I and T119N mutant groups were likely to have evolved to become distinct sub-populations of DENV-2 cosmopolitan clade III associated with mild disease.

## Discussion

The present study was carried out during a period (July 2010-January 2013) of DENV-2 dominance [19] in Singapore that commenced subsequent to an outbreak in 2007 [33]. DENV-2 contributed to more than 70% of indigenous cases serotyped from 2007 to 2011[19]. The outbreak in 2007 was caused by clade II of DENV-2 cosmopolitan genotype. The empirical evidence suggests that clade III strains analyzed in the present study descended from those of clade II [34] and remained as the dominant DENV strain from 2010 to 2012 [19, 33–35]. Unlike the period of clade II, dengue incidence remained stable without notable epidemics during the period of clade III’s dominance, suggesting that the evolution of DENV-2 clade II into clade III presumably resulted in relatively a less-virulent virus population. Our findings of two groups of clade III mutants (NS2A-V83I and NS2A-T119N) that were significantly associated with mild disease (DF) supported this assumption.

### Structural and functional significance of amino acid differences in NS2A-V83I and NS2A-T119N mutants

Flavivirus NS2A is a 22 KDa hydrophobic protein [36] that interacts with 3’ UTR, NS3 and NS5 proteins in the replication complex at the host-cell endoplasmic reticulum (ER) membrane [37]. Besides its involvement in flavivirus replication, NS2A plays a role in virion assembly [38, 39] and the inhibition of interferon signaling (28). Several previous studies suggested a critical role for NS2A protein in the attenuation of flavivirus virulence [40–43]. Therefore, it was noteworthy that both groups of DENV-2 that were exclusively associated with mild disease outcome in our patient cohort possessed signature substitutions in NS2A protein (NS2A-V83I and NS2A-T119N) (Fig. 2).

The first group of isolates (n = 15) possessed their signature mutation at residue 83 of NS2A protein (NS2A-V83I) (Fig. 2). According to the model proposed by Xie and colleagues, residue NS2A-83 resides in the pTMS3 trans-ER membrane domain [44]. The pTMS3 domain consists of two α-helices separated by a helix “breaker” region spanning from residues 83 to 85 [44]. Mutations at residue 84 (R84E and R84A), but not 85, have been shown to attenuate DENV-2 RNA synthesis and infectious virion formation [44]. Moreover, Steel et al (2010) also reported a mutation at residue NS2A-83 (NS2A-S83G) potentially associated with natural attenuation of DENV-2 in Tonga [45]. Therefore, empirical evidence indicates that NS2A-83 resides in a domain that has a potential role in the attenuation of DENV-2, implying a genetic basis for the association between NS2A-V83I mutants and mild disease outcome observed in our patient cohort. However, the fact that NS2A-V83I is a conservative change may jeopardize this assumption.

Alternatively, the less virulent phenotype observed in NS2A-V83I mutants may be due to specific interactions between NS2A-V83I substitution and the rest of coexisting amino acid substitutions fixed in all isolates (C-P43T+NS1-S103T+NS3-R337K) and sub-groups (NS3-I600T+ NS5-P136S) of the mutant group. Of them, C-43 is a highly conserved non-polar, hydrophobic residue with proline in many medically important mosquito-borne flaviviruses [46]. The deletion of residues 41–44 of capsid protein is known to attenuate DENV-2 with lower virus titers and less neurovirulence than a wild-type virus in mice [47]. Being a polar and hydrophilic change, C-P43T substitution in NS2A-V83I mutants is, therefore, likely to be of structural significance and may contribute to less virulent clinical outcome of infections due to NS2A-V83I mutants. DENV NS1 is a homologous protein that exists either in bound or secreted forms [48–50]. While the intracellular bound form is believed to play an essential role in virus replication [51], secreted and cell-surface associated NS1 has been implicated in protection, immune evasion and pathogenesis [52]. Evidence so far has identified three structural domains of flavivirus NS1 [52, 53]. Domain I (residues 1–157) is evidently the most immuno-dominant [53]. There are 12 conserved cysteine residues distributed across the whole polypeptide and two conserved N-linked glycosylation sites (residues 130 and 207) in domains I and II (residues 158–235) that play an essential role in virus replication [52–54]. Even though NS1-S103T resides within the most immuno-dominant domain of NS1, there is no previous evidence to suggest any structural and functional significance of residue 103. NS3 protein carries helicase and protease properties and therefore, is an essential element of flavivirus replication and polyprotein processing [55]. NS3-337 resides in domain II of the helicase region of NS3 protein which is involved in virus replication [56]. In DENV-2, arginine is highly conserved at NS3-337 within a cluster of charged amino acids spanning from 334–338 (DEERE). Previous evidence has shown that mutagenesis of charged residues at 334–336 restricts the replication efficiency of DENV-2 [57]. However, there is lack of evidence regarding any specific role of residues 337 and 338 of DEERE domain on virus replication efficiency. Nevertheless, being conservative alterations, both NS1-S103T and NS3-R337K substitutions are unlikely to have substantial structural implications on NS2A-V83I mutants.

NS3-I600T, on the other hand, is a non-conservative change that resides at the C-terminal of domain III of DENV helicase [56]. A recent study reported that the C-terminal of DENV helicase domain III interacts with NS5 during virus replication [58]. The interactive domain of NS3 was mapped to amino acids 566–585, with 570 having a critical role in replication efficiency [58]. Another study reported that DENV-2 NS4B also interacts with helicase domains II and III during virus replication [51]. Moreover, residues 601and 603 in domain III have been shown to stabilize the contacts between domain I and domain III of Yellow Fever virus helicase [59]. Nevertheless, any structural or functional significance of residue NS3-600 in DENV is currently unknown. NS5 is the largest flaviviral protein with its N-terminal domain (residues 1–265) having methyltransferase (MTase) activity and C-terminal domain (residues 273–900) acting as the RNA dependent RNA polymerase (RdRp) [60]. NS5-P136S observed in NS2A-V83I mutants is a non-conservative substitution that resides in the core domain (residues 59–224) of MTase region [60]. Although the hydrophobic to hydrophilic transition of NS5-P136S, with an alteration to a H-bonding amino acid, may have structural implications, residue 136 does not fall within important functional motifs of MTase domain such as K-D-K-E motif, S-adenosyl-L-methionine binding pocket, GTP-binding site and RNA binding site [61, 62].

The second group of NS2A mutants (n = 6) that were exclusively associated with a mild disease outcome in our study, possessed NS2A-T119N substitution. NS2A-119 is a hydrophilic residue exposed within the ER lumen during DENV replication [44]. Although any direct functional significance of NS2A-119 is unclear, Audsley *et al* (2011) showed that A30P substitution, together with substitutions at six other residues including 119 of NS2A protein, contributed to a dramatic reduction in the virulence and cytopathicity of West Nile virus [42]. NS2A-T119N substitution has also been reported in six DENV-2 (American Asian genotype) isolates from Nicaragua. Only 2 of them were clinically classified and similar to our findings, both did not suffer from the severe disease [63].

### Nucleotide differences in UTRs of NS2A-V83I mutants altered the structure and potentially impaired virus replication

**Fig 3:**
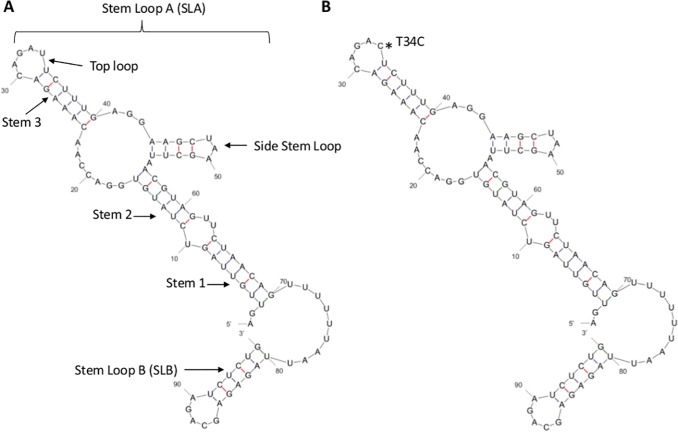
Comparison of RNA secondary structures of the variable region of 3’UTR between wild type (NS2A-V83) and mutant (NS2A-V83I) variants. The first (5’ end) 120 nucleotides of 3’UTR was used to draw the secondary structure as predicted by the MFOLD web server [31]. Position 1 indicates the first nucleotide of 3’UTR. (A). Wild type (NS2A-V83) DENV-2 cosmopolitan clade III (B). Mutant (NS2A-V83I) DENV-2 cosmopolitan clade III. The mutated position (19T) in Stem Loop-I (SL-I) is indicated with an asterisk. Stem Loop structures (I-III) were named as illustrated elsewhere [68].

Besides the coding genome, flavivirus UTRs are also important elements of RNA synthesis and virus replication [64]. The 96-nucleotide region of DENV-2 5’UTR includes two main domains; a large stem loop (SLA) and a short stem loop (SLB) [64]. SLA structure is believed to interact with the RNA dependent RNA polymerase domain of NS5 protein during DENV replication [65]. SLB, on the other hand, holds the 5′ upstream AUG region (UAR) that interacts with 3’UTR in a long-distance RNA-RNA interaction which unwinds the terminal 3’ stem loop during the initiation of DENV RNA synthesis [66, 67]. The SLA structure contains three helical regions, a top loop and a side stem loop. The T34C transition observed in 5’ UTR of NS2A-V83I mutants resides within the top loop of SLA structure, without altering the predicted structure (Fig. 3). Nucleotide substitutions at 30–34 positions of the top loop have previously been shown to affect the polymerase activity in a sequence-dependent way, thereby impairing DENV replication [65].

**Fig 4:**
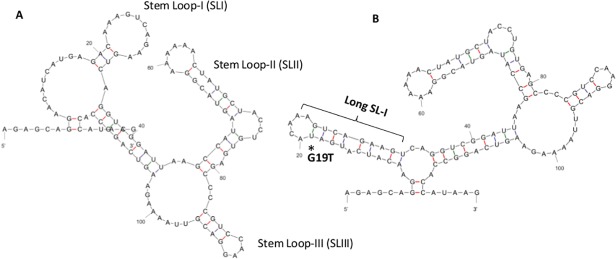
Comparison of RNA secondary structures of the 5’UTR between wild type (NS2A-V83) and mutant (NS2A-V83I) variants. The complete sequence (96 nucleotides) of 5’UTR was used to draw the secondary structure as predicted by the MFOLD web server [31]. Position 1 indicates the first nucleotide of 5’UTR. (A). Wild type (NS2A-V83) DENV-2 cosmopolitan clade III (B). Mutant (NS2A-V83I) DENV-2 cosmopolitan clade III. The mutated position (34C) in the top loop of Stem Loop A (SLA) is indicated with an asterisk. Stem Loop structures were named as illustrated elsewhere [64].

The approximately 450 nucleotide long DENV 3’UTR is divided into three domains; hyper-variable, semi-variable and conserved domains [64]. The critical functional elements of viral replication are located in the conserved domain, whereas semi-variable and hyper-variable regions act as enhancers of replication [64]. Long-range deletions in the hyper-variable region have been shown to decrease DENV replication in mammalian cells [69]. As shown in Fig. 4, the A19T transversion in NS2A-V83I mutants resulted in a relatively long stem of the stem loop I (SL-I) structure of the hyper-variable region [68], of which the function has not yet been clearly elucidated. In overall, the analysis of mutation profiles suggested the likelihood of impaired replication efficiency of NS2A-V83I and NS2A-T119N mutants. As the virus titer is known to positively correlate with disease severity [11], it is of interest to scientifically prove that these genetic and configuration changes in UTRs, together with additional substitutions in the coding region, alter the replication efficiency of NS2A-V83I and NS2A-T119N mutants, thereby resulting in a mild clinical outcome as observed in our study.

### Structural and functional significance of substitutions detected in individual and small groups of isolates

Of 39 amino acid substitutions detected, 29 substitutions were found either in individual or small groups (2–3 isolates) of isolates (Table 2). Of them, there were amino acid substitutions specifically detected in viruses isolated from DHF (E-K36R, E-V197A, E-V250I, E-P364T, NS5-P583Q, NS5-V725A and NS5-R891I) and DF (C-R82K, E-I322V, E-V428M, NS2A-T42I, NS2A-L53V, NS2A-Q113H, NS2A-E118K, NS3-K170E, NS3-S171N, NS5-E169D, NS5-K548E, NS5-A648T, NS5-S763N, NS5-D808N and NS5-P829L) cases. Of these, only E-P364T, NS2A-T42I, NS5-P583Q, NS5-A648T and NS5-R891I substitutions are non-conservative. As shown in Table 2, the majority of above substitutions were under purifying selection, indicating a potential fitness cost on respective isolates.

In flaviviruses, N-terminal capsid sequence encodes for essential functional domains of virus replication such as 5’ cyclization sequence [70], hairpin element [71], 5’ downstream of AUG region [67] and C1/C2 regions [72] as well as an intracellular membrane-associated internal hydrophobic region (residues 46–66) [73, 74]. C-R82K substitution does not reside within any of these known domains. DENV E protein consists of three domains; EDI, EDII (collectively amino acid (aa) residues 1–294) and EDIII (295–392 aa) [75, 76]. Six E protein substitutions mentioned above are distributed in all three domains of E protein; E-K36R (EDI), E-V197A and E-V250I (EDII) and E-I322V, E-V347A and E-P364T (EDIII) as well as the transmembrane anchor (E-V428M) [75, 76]. None of them resided within known functional domains such as fusion loop, ligand-binding pocket at the interphase between EDI and II [75], neutralization epitopes [77, 78] and virulent motifs [16]. However, the dominant presence of E protein mutations in isolates obtained from DHF cases warrants their further evaluation to determine a potential role in pathogenicity. On the other hand, even though not associated with known functional motifs, the distribution of all NS2A substitutions among individual and small groups of isolates causing DF cases was also noteworthy. Flavivirus NS2A has previously been implicated in flavivirus assembly [39], release of infectious virus particles [38] and less severe pathogenicity [42, 45]. NS3-K170E and NS3-S171N substitutions reside at the C-terminal end of the protease domain [55] and do not hold any known functional significance. The highest number of mutations among individual and small groups of isolates was detected in NS5 protein. NS5 is the largest protein in DENV genome that has shown a high mutation rate among clinical isolates [63]. Except for NS5-E169D, all NS5 mutations listed in this section resides within the RdRp domain that is responsible for the virus replication through *de-novo* RNA synthesis [55]. Of them, NS5-P583, NS5-V725, NS5-K548, NS5-D808 and NS5-P829 residues are conserved in DENV-2. Notably, NS5-D808N is in the priming loop (residues 782–809) that forms the upper part of the RNA template tunnel and regulates the movement of template RNA during virus replication [79]. In addition, NS5-V725A is in a strictly conserved motif of the thumb domain of flavivirus RdRp [79]. These observations warrant further studies to determine any role of latter substitutions in DENV replication.

### Association of amino acid differences with known B- and T-cell epitopes

Host immune response is considered to be an important factor that shapes the clinical outcome of DENV infections. Both T- and B-cell responses play a role in this process through production of cytokines and virus neutralizing antibodies [80, 81]. It has been postulated that preferential activation of memory T lymphocytes with lower avidity for the infecting virus (‘original antigenic sin’) in secondary DENV infections result in altered T-cell functional responses that lead to plasma leakage in DHF/DSS [82]. Another phenomenon is that poorly neutralizing, low-avidity cross-reactive antibodies exacerbate disease by facilitating virus entry into cells bearing Fcγ receptors (‘antibody dependent enhancement’), resulting in high viral titres and severe disease in secondary infections [83–86]. However, association between the magnitude of T-cell response and disease severity remains inconclusive [87, 88]. Instead, recent evidence suggests a more protective role of the T-cell response in DENV infections than its involvement in severe disease [81, 89].

Empirical evidence has shown that the specificity of T-cell response against DENV is broad, but primarily directed against the NS3 and NS5 proteins [87–91]. Besides, C, prM, E, NS1, NS2A, NS4a and NS4b proteins of the virus have also been indicated as regions recognized by CD4+ and CD8+ T cells [87, 91–95]. DENV proteome-based peptide screening has identified numerous epitopes that substantially evoke T-cell response [87, 91]. However, none of the substitutions (C-P43T, E-V164I, NS1-S103T, NS2A-V83I, NS2A-T119N, NS2A-L153S and NS5-P136S), except for NS3-R337K and NS3-I600T, detected in three main virus groups described in the present study resided within known T-cell epitope regions. NS3-337 resides within a known CD8+ T-cell epitope that has been shown to enhance IFN-γ production in HLA type B*5801 individuals [91]. Similarly, NS3-I600T has also been shown to be within an immunogenic CD8+ epitope in HLA type A*2402 individuals [91]. On the other hand, NS2A-L53V, NS3-A558V, NS5-E169D and NS5-S763N substitutions observed either as singletons or in small groups (Table 2) have also been reported to constitute CD8+ epitopes in DENV-2 [91]. Moreover, C-R82K and E-V250I substitutions fall within known CD4+ epitopes [87, 91]. A strong interferon response has been shown to correlate with mild clinical manifestations due to DENV infections [96]. As the majority of T-cell epitope-related substitutions, especially NS3-R337K, were detected in patients who developed mild disease (Table 2), further investigations are needed to determine the role of them in innate immune response to DENV.

Likewise, the specificity of humoral immune response to DENV also appears broad. Antibodies to C, prM, E, NS1, NS3, NS4a and NS4b proteins have previously been detected in DENV-infected patient sera and epitope mapping has identified several immunogenic regions in respective proteins [97, 98]. Being DENV surface proteins, E and prM proteins are the primary targets of the immune response. Of these, E protein is the principal target of neutralizing mouse monoclonal antibodies (MAb) [99, 100]. While all three domains (EDI-EDIII) of E protein contain cross-reactive, predominantly non-neutralizing epitopes, most strongly neutralizing MAbs bind to epitopes in EDIII [77, 78, 101–106]. Important epitopes identified on prM are mapped to aa 53–67 [107] and aa 40–49 [108] and antibodies against both epitopes have been shown to cross react with epitopes in EDII and hinge region between EDI and EDII of DENV-2 [108, 109]. Moreover, antibodies bound to both epitopes have been shown to produce highly infectious immature DENV particles, therefore potentially contributing to the development of severe disease [110]. Of eight E protein substitutions detected in this study (Table 2), four substitutions (E-K36R, E-V164I, E-V197A and E-V250I) were in EDI-II and three substitutions (E-I322V, E-V347A and E-P364T) were in EDIII. Of them, E-K36R, E-V197A, E-V250I and E-P364T were found either as singletons or in combination with remaining E protein substitutions in individual patients who manifested severe (DHF) disease (S1 Table). However, none of those substitutions were located in known B-cell epitopes [77, 78, 101].

On NS1, previous studies have revealed five immuno-dominant B-cell epitopes (1–15, 21–35, 111–125, 191–205, and 261–275 aa), which bind DENV type-specific and group-specific antibodies [111–114]. On C and NS4a proteins, several DENV type-specific and cross-reactive epitopes (C: 2–10, 82–85 and 91–99 aa; NS4a: 17–22, 29–34 and 41–46 aa) have been described [97]. Interestingly, residues 82–85 in C protein have also been identified as a CD4+ T-cell epitope [91] and C-R82K substitution detected in an individual DF infection of the present study resides within this motif (S1 Table). A phage-display random peptide library analysis by Amin and colleagues showed potential B-cell peptides on NS3 (425–432 and 537–544 aa) and NS4b (161–172 aa) [98]. However, none of NS1 and NS3 substitutions reported in the present study constitute known B-cell epitopes.

## Conclusions

Our findings indicated that subtle genetic differences in a highly homogenous, monotypic DENV-2 population potentially modified the clinical outcome of our study subjects, regardless of primary and secondary infection status. Interestingly, none of the fixed genetic differences in our study isolates was specifically associated with a severe disease outcome. This observation indicated that disease progression into DHF and DSS in our patient cohort was more likely to be due to host than virus factors. However, our observations were made in relatively a small group of patients and further investigations are required, preferably in large cohorts, to determine how the observed genetic differences regulate virus replication and modulate immune response in infected individuals. It should also be noted that we did not explore variations in the strain-dependent immune reactions against different groups of virus strains within our patient cohort that can potentially influence the clinical outcome through heterotypic antibody responses [115, 116]. Moreover, we did not analyze the quasispecies nature of our study virus population. What we have assessed here are the genetic differences in consensus sequences at the inter-host level rather than those within individual hosts. Even though the concept of intra-host diversity has been proven in DENV populations [63], its relationship with disease severity remains controversial [63, 117, 118]. While further investigations are necessary to determine whether substitutions observed in NS2A-V83I and NS2A-T119N mutant groups truly cause virus attenuation resulting in mild disease, we hypothesize that their fixation within a highly homogenous virus population associated with a less-virulent disease outcome is an evolutionary adaptation for better survival within the human-mosquito transmission cycle of DENV. This warrants future work to determine the behavior of distinct groups of DENV-2 observed in this study in mammalian and *Ae*. *aegypti* hosts, the primary vector of DENV, in order to decipher the role of DENV diversity in shaping dengue epidemiology.

## Supporting Information

S1 Table Details of isolates with amino acid substitutions, clinical outcome and infection status within the DENV-2 cosmopolitan clade III cohort. Isolate IDs in bold letters are dengue hemorrhagic fever (DHF) cases. Those in bold and underlined are dengue shock syndrome (DSS) cases. £Infection status is indicated in brackets; 10 = primary, 20 = secondary, DF = dengue fever.
(DOC)
