# Supplementary material for: Clinical Outcome and Genetic Differences within a Monophyletic Dengue Virus Type 2 Population
Source: PLoS One. 2015 Mar 26;10(3):e0121696. doi: 10.1371/journal.pone.0121696 (PMC4374945; doi:10.1371/journal.pone.0121696)
Supplement: S1 Table — Isolate IDs in bold letters are dengue hemorrhagic fever (DHF) cases. Those in bold and underlined are dengue shock syndrome (DSS) cases. £Infection status is indicated in brackets; 10 = primary, 20 = secondary, DF = dengue fever. (DOC) [file pone.0121696.s001.doc]

**Table S1. Details of isolates with amino acid substitutions, clinical outcome and infection status within the DENV-2 cosmopolitan clade III cohort**

| **Substitution** | **No. of isolates** | **DF/DHF/DSS** | **Isolate ID/s and infection status^£^** |
| --- | --- | --- | --- |
| C-P43T | 19 | DF, n=17  DHF, n=1  DSS, n=1 | DC648 (2^0^), DC719 (2^0^), DC759 (1^0^), DC792 (1^0^), **DC793** (2^0^), DC795 (1^0^), DC796 (2^0^), DC802 (2^0^), **DC814** (2^0^), DC827 (1^0^), E1379 (2^0^), E1429 (1^0^), E1432 (1^0^), E1433 (1^0^), E1434 (1^0^), E1436 (2^0^), E1439 (2^0^), E1502 (1^0^), E1519 (2^0^) |
| C-R82K | 1 | DF | DC412 (2^0^) |
| E-K36R | 1 | DHF | **DC378** (2^0^) |
| E-V164I | 9 | DF, n=5  DHF, n=4 | DC730 (1^0^)**, DC735** (1^0^), **DC756** (1^0^), DC763 (2^0^), DC766 (2^0^), **DC786** (1^0^), DC790 (2^0^), DC811 (1^0^), **DC812** (2^0^) |
| E-V197A | 1 | DHF | **DC408** (2^0^) |
| E-V250I | 1 | DHF | **DC812** (2^0^) |
| E-I322V | 1 | DF | DC412 (2^0^) |
| E-V347A | 3 | DF, n=1  DHF, n=2 | DC331 (1^0^), **DC378** (2^0^), **DC389** (1^0^) |
| E-P364T | 1 | DHF | **DC391** (2^0^) |
| E-V428M | 2 | DF, n=2 | DC645 (2^0^), DC649 (2^0^) |
| NS1-S40P | 2 | DF, n=1  DHF, n=1 | DC353 (1^0^), **DC410** (2^0^) |
| NS1-S103T | 19 | DF, n=17  DHF, n=1  DSS, n=1 | DC648 (2^0^), DC716 (1^0^), DC759 (1^0^), DC792 (1^0^), **DC793** (2^0^), DC795 (1^0^), DC796 (2^0^), DC802 (2^0^), **DC814** (2^0^), DC827 (1^0^), E1379 (2^0^), E1429 (1^0^), E1432 (1^0^), E1433 (1^0^), E1434 (1^0^), E1436 (2^0^), E1439 (2^0^), E1502 (1^0^), E1519 (2^0^) |
| NS1-V286A | 3 | DF, n=1  DHF, n=2 | DC771 (2^0^), **DC848** (2^0^), **DC849** (2^0^) |
| NS2A-T42I | 1 | DF | DC429 (1^0^) |
| NS2A-L53V | 1 | DF | E1379 (2^0^) |
| NS2A-V83I | 15 | DF, n=15 | DC648 (2^0^), DC719 (2^0^), DC795 (1^0^), DC796 (2^0^), DC802 (2^0^), DC827 (1^0^), E1379 (2^0^), E1429 (1^0^), E1432 (1^0^), E1433 (1^0^), E1434 (1^0^), E1436 (2^0^), E1439 (2^0^), E1502 (1^0^), E1519 (2^0^) |
| NS2A-Q113H | 1 | DF | DC673 (1^0^) |
| NS2A-E118K | 1 | DF | DC589 (2^0^) |
| NS2A-S119N | 6 | DF, n=6 | DC348 (1^0^), DC380 (2^0^), DC403 (1^0^), DC427 (2^0^), DC429 (1^0^), DC430 (1^0^) |
| NS2A-L153S | 10 | DF, n=5  DHF, n=5 | **DC621** (2^0^), DC730 (1^0^), **DC735** (1^0^), **DC756** (1^0^), DC763 (2^0^), DC766 (2^0^), **DC786** (1^0^), DC790 (2^0^), DC811 (1^0^), **DC812** (2^0^) |
| NS3-K170E | 2 | DF, n=2 | DC641 (2^0^), DC654 (2^0^) |
| NS3-S171N | 1 | DF | DC792 (1^0^) |
| NS3-R337K | 20 | DF, n=17  DHF, n=2  DSS, n=1 | **DC367** (2^0^), DC648 (2^0^), DC719 (2^0^), DC759 (1^0^), DC792 (1^0^), **DC793** (2^0^), DC795 (1^0^), DC796 (2^0^), DC802 (2^0^), **DC814** (2^0^), DC827 (1^0^), E1379 (2^0^), E1429 (1^0^), E1432 (1^0^), E1433 (1^0^), E1434 (1^0^), E1436 (2^0^), E1439 (2^0^), E1502 (1^0^), E1519 (2^0^) |
| NS3-A558V | 3 | DF, n=2  DHF, n=1 | DC661 (1^0^), **DC666** (2^0^), DC716 (1^0^) |
| NS3-I600T | 4 | DF, n=4 | E1429 (1^0^), E1432 (1^0^), E1433 (1^0^), E1436 (2^0^) |
| NS5-P136S | 4 | DF, n=4 | DC795 (1^0^), DC796 (2^0^), DC802 (2^0^), DC827 (1^0^) |
| NS5-E169D | 1 | DF | DC412 (2^0^) |
| NS5-A196T | 2 | DF, n=1  DHF, n=1 | DC353 (1^0^), **DC410** (2^0^) |
| NS5-K548E | 1 | DF | DC802 (2^0^) |
| NS5-P583Q | 1 | DHF | **DC357** (2^0^) |
| NS5-N645D | 3 | DF, n=2  DSS, n=1 | DC759 (1^0^), DC792 (1^0^)**,** **DC814** (2^0^) |
| NS5-I648T | 1 | DF | DC802 (2^0^) |
| NS5-V725A | 1 | DHF | **DC367** (2^0^) |
| NS5-S763N | 1 | DF | DC412 (2^0^) |
| NS5-A778S | 3 | DF, n=3 | DC710 (2^0^), DC720 (2^0^), DC740 (1^0^) |
| NS5-D808N | 1 | DF | DC619 (2^0^) |
| NS5-P829L | 1 | DF | DC649 (2^0^) |
| NS5-S832T | 3 | DF, n=1  DHF, n=2 | DC331 (1^0^), **DC378** (2^0^), **DC389** (1^0^) |
| NS5-R891I | 1 | DHF | **DC786** (1^0^) |

Isolate IDs in bold letters are dengue hemorrhagic fever (DHF) cases. Those in bold and underlined are dengue shock syndrome (DSS) cases. DF=dengue fever

^£^Infection status is indicated in brackets; 1^0^=primary, 2^0^=secondary
